# Vaccinomics to Design a Multi-Epitopes Vaccine for *Acinetobacter baumannii*

**DOI:** 10.3390/ijerph19095568

**Published:** 2022-05-04

**Authors:** Miraj ud-din, Aqel Albutti, Asad Ullah, Saba Ismail, Sajjad Ahmad, Anam Naz, Muhammad Khurram, Mahboob ul Haq, Zobia Afsheen, Youness El Bakri, Muhammad Salman, Bilal Shaker, Muhammad Tahir ul Qamar

**Affiliations:** 1 Department of Health and Biological Sciences, Abasyn University, Peshawar 25000, Pakistan; mirajkhattak193@gmail.com (M.u.-d.); asadullahaup@gmail.com (A.U.); zobia.afsheen@abasyn.edu.pk (Z.A.); muhammad.salman@abasyn.edu.pk (M.S.); 2 Department of Medical Biotechnology, College of Applied Medical Sciences, Qassim University, Buraydah 52571, Saudi Arabia; 3 Department of Biological Sciences, National University of Medical Sciences, Rawalpindi 46000, Pakistan; sabaismail7@gmail.com; 4 Institute of Molecular Biology and Biotechnology (IMBB), The University of Lahore, Lahore 54590, Pakistan; anam.naz88@live.com; 5 Department of Pharmacy, Abasyn University, Peshawar 25000, Pakistan; muhammad.khurram@abasyn.edu.pk (M.K.); dr.mahboobulhaq@gmail.com (M.u.H.); 6 Department of Theoretical and Applied Chemistry, South Ural State University, Lenin Prospect 76, 454080 Chelyabinsk, Russia; yns.elbakri@gmail.com; 7 Department of Biomedical Engineering, Chung-Ang University, Seoul 06974, Korea; ch.bilal321@outlook.com; 8 Department of Bioinformatics and Biotechnology, Government College University Faisalabad, Faisalabad 38000, Pakistan; tahirulqamar@gcuf.edu.pk

**Keywords:** *Acinetobacter baumannii*, pan-genomics, core genomics, epitope vaccine, molecular dynamics simulations

## Abstract

Antibiotic resistance (AR) is the result of microbes’ natural evolution to withstand the action of antibiotics used against them. AR is rising to a high level across the globe, and novel resistant strains are emerging and spreading very fast. *Acinetobacter baumannii* is a multidrug resistant Gram-negative bacteria, responsible for causing severe nosocomial infections that are treated with several broad spectrum antibiotics: carbapenems, β-lactam, aminoglycosides, tetracycline, gentamicin, impanel, piperacillin, and amikacin. The *A. baumannii* genome is superplastic to acquire new resistant mechanisms and, as there is no vaccine in the development process for this pathogen, the situation is more worrisome. This study was conducted to identify protective antigens from the core genome of the pathogen. Genomic data of fully sequenced strains of *A. baumannii* were retrieved from the national center for biotechnological information (NCBI) database and subjected to various genomics, immunoinformatics, proteomics, and biophysical analyses to identify potential vaccine antigens against *A. baumannii*. By doing so, four outer membrane proteins were prioritized: TonB-dependent siderphore receptor, OmpA family protein, type IV pilus biogenesis stability protein, and OprD family outer membrane porin. Immuoinformatics predicted B-cell and T-cell epitopes from all four proteins. The antigenic epitopes were linked to design a multi-epitopes vaccine construct using GPGPG linkers and adjuvant cholera toxin B subunit to boost the immune responses. A 3D model of the vaccine construct was built, loop refined, and considered for extensive error examination. Disulfide engineering was performed for the stability of the vaccine construct. Blind docking of the vaccine was conducted with host MHC-I, MHC-II, and toll-like receptors 4 (TLR-4) molecules. Molecular dynamic simulation was carried out to understand the vaccine-receptors dynamics and binding stability, as well as to evaluate the presentation of epitopes to the host immune system. Binding energies estimation was achieved to understand intermolecular interaction energies and validate docking and simulation studies. The results suggested that the designed vaccine construct has high potential to induce protective host immune responses and can be a good vaccine candidate for experimental in vivo and in vitro studies.

## 1. Introduction

Antibiotic resistance or AR refers to the resistance ability of bacteria to the action of antibiotics to which they were susceptible before. In the last 25 years, bacteria have developed AR very rapidly, and the situation is alarming now [1]. Resistance is rising to a high level across the globe, and novel resistant strains are emerging and spreading very fast [2]. This process occurs due to the evolution of novel mutations in the bacterial genome or acquisition of resistant genes from the environment. Misuse and overuse of antibiotics also contributed significantly to AR [3]. AR genes are transferred from one bacterium to the other through plasmid, which disseminates the resistant genes within/between the species [4]. Some bacteria are resistant to one type of antibiotic while others are multi-drug resistant (MDR), extensive drug resistant (XDR) [5], and pan drug resistant, showing resistance to multiple, many, and all classes of antibiotics, respectively [6]. The number of infections and deaths that occur due to AR is very high. Every year, more than 50,000 deaths occur in America and Europe only due to Methicillin-resistant *Staphylococcus aureus* (MRSA) [7]. Tuberculosis is still affecting developing countries and leads to a high number of deaths every year [8]. It is tough to manage AR bacterial infections according to the center for disease and control (CDC) survey [9]. Approximately 2.8 million bacterial infections are reported each year, of which more than 35,000 lead to deaths. This means that every 15 min, 1 person dies due to AR [10]. The situation is equally bad all over the world, so immediate actions are needed to combat AR [11]. According to a report by the World Health Organization (WHO), if the situation is not handled properly AR may lead to 10 million deaths and an economic loss of more than 100 trillion dollars every year [12]. In conclusion, we are heading towards the post-antibiotic era, which warrants the introduction of novel therapeutic strategies to address AR health crises [13].

To overcome the burden of AR, many strategies can be applied. This can be achieved by introducing new methodologies that can help in identifying new drug targets and new classes of antibiotics [14]. Traditional technologies of drug target identification and drug development are very slow and costly [15]. Advanced sequencing technologies allow a deeper understanding of bacterial biology and deciphering metabolic processes [16]. This opens up new avenues for developing drugs to manage super drug resistant species that it was not possible to manage before. One way to stop the evolution of AR is the development of antivirulent compounds [16,17]. These compounds only interfere with the pathogenic and virulent attributes of the pathogen but do not force bacteria to change their genome in response to the action of the compound. This is exemplified by the inactivation of quorum sensing signal molecules (through a process known as QS inhibition or quorum quenching (QQ)) to stop bacterial biofilm formation and multiple virulence factors regulators [18,19]. This can be accomplished in several ways, such as through the development of drugs against QS signal molecules and the enzymatic blockage of QS signal molecules pathways. Quorum sensing inhibitors can be used as an alternative to antibiotics [8].

A vaccine is a biological preparation that provides protection against harmful diseases. Vaccination is the most effective method of preventing infections [20]. Historically, vaccines for managing and preventing the spread of AR bacterial pathogens have been underrated, but it is a well-established method to tackle the AR pathogen [21]. For example, *Strep pneumoniae* (pneumococcal) conjugate and *Hemophilus influenza* type B (Hib) vaccines are praised for their ability to provide protection against the aforementioned pathogens and lower the use of antibiotics against them [22]. Considering this, vaccines have the potential to provide a permanent solution to tackle AR bacteria. *A. baumannii* is a Gram-negative bacteria usually found in hospital environments. It causes a variety of nosocomial infections, such as pneumonia, meningitis, wound infections, and urinary tract infections (UTIs), which are difficult to control and prevent [23]. *A. baumannii* is treated with several broad-spectrum antibiotics: carbapenems, β-lactam, aminoglycosides, tetracycline, gentamicin, imipenem, piperacillin, and amikacin [10]. Over the last 15 years, this bacteria has been a high risk to human health because it has acquired resistance to most antibiotics and has become a serious health threat [24,25]. No vaccine is currently available against this pathogen, which makes the situation more worrisome [26]. Therefore, efforts are needed to identify protective antigenic peptides from the core genome of the pathogen, which can easily be analyzed in experimental studies. Findings of the study will save time and may lead to cost-effective vaccine development.

## 2. Research Methodology

**Figure 1:**
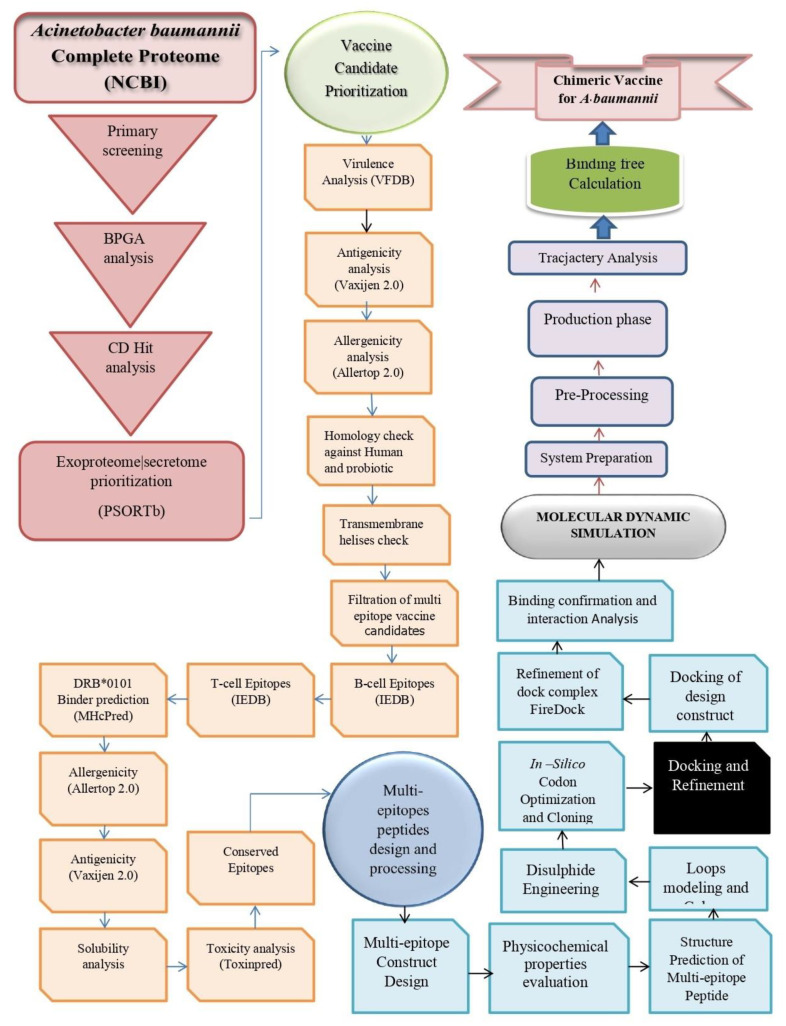
Schematic framework of the methodology used in this study.

The study was initiated by retrieving complete sequenced genomes of *A. baumannii* from the national center for biotechnological information (NCBI) database. Subtractive proteomic and reverse vaccinology (RV) techniques were applied to prioritize vaccine candidates against *A. baumannii*. This whole process was completed in three phases. The step wise procedure applied herein is presented in Figure 1.

### 2.1. Complete Genome Retrieval

The complete sequenced genome of *Acinetobacter baumannii* (~50 in number) was retrieved from NCBI database. The core and dispensable genomes were identified using bacterial pan-genome analysis [24].

### 2.2. Pre-Screening Phase

The primary phase was named as a pre-screening phase. During this phase, antigenic and virulence factor molecules were selected. Those proteins were selected that (i) showed sequence conservation among all sequenced genomes [27], (ii) were not similar to the human proteome as they may generate autoimmune responses [28], (iii) were present on the surface of the pathogen because of their strong ability to stimulate the host immune system [29], (iv) were essential and crucial for the survival of the pathogen due to their key cellular functions [30], (v) were part of the core proteome [31], and (vi) were non-redundant [32], because redundant proteins are not part of the core genome and are poor immunological targets [33]. On the other hand, non-redundant proteins are good vaccine candidates because they are present in all strains and have vital functions in cells [34]. The redundancy of proteins was predicted through a server called Cd-Hit [35] by considering a sequence identity threshold of 50%. Then, homology of the proteins was checked against the human proteome through an online tool named BlastP [36]. There are different parameters for identifying the similarity between human and bacterial proteins. Proteins with less than 30% sequence identity, a bit score of 100 or more, and an E-value smaller than 1.0 × 10^−4^ [37] were regarded as host non-homologous. Another server, PsortB, was used to predict the surface localized proteins [38,39]. Only extracellular, periplasmic, and outer membrane localized proteins were subjected to further analysis, and all the cytoplasmic membrane proteins were discarded [40].

### 2.3. Vaccine Epitopes Prioritization Phase

During this phase, further filtration of the pathogen secretome and exoproteome was conducted. Virulent analysis was carried out to check the involvement of proteins in the pathogen infection pathway [41]. This selection was done via BlastP against the virulent factor database (VFDB) [42]. Proteins (virulence factors) with 30% identity and a bit score higher than 100 were chosen [43]. The physiochemical properties of the selected virulence factors were checked via Protparam [44]. Only proteins that were easy to use in experimental analysis were selected [45]. Proteins with an instability index of less than 40 were selected, and those with more than 40 were marked unstable. The proteins were then analyzed for their molecular weight; those with a molecular weight of less than 110 kDa were considered as the best vaccine candidates; HMMTOP and THMM servers were then used to examine the number of transmembrane helices [46]. Only proteins with 0 and 1 transmembrane helices were selected for further processing [24,29]. The prediction of antigenicity was performed using Vaxijen2.0 [47]. The adhesive nature of the selected antigenic proteins was predicted using another server called SPAAN with a minimum value of 0.5 [48]. To overcome the chances of inhibiting beneficial bacteria, the sequence alignment of filtered proteins was performed out with probiotic bacterial proteomes [49]. For this, 4 lactobacillus bacterial species (*L. rhamnosus* (taxid: 47,715), *L. casei* (taxid: 1582), *L. gasseri* (taxid: 1596) and *L. johnsonii* (taxid: 33,959)) and one Bifidobacterium specie, i.e., *B. bifidum* (taxid: 1681), were selected, and a BlastP search was accomplished [50]. Furthermore, the similarity of proteins was checked against mouse proteome just to extract those proteins that were showing no homology to the mouse proteins. This was performed to decrease the chances of autoimmune reactions and reduce false positive results during trails testing of the vaccine candidate [51]. After passing through these filters, the selected proteins were predicted for B-cell and T-cell epitopes [52,53,54]. Linear B cell prediction was performed first using the IEDB server [54]. T-cell prediction was performed on the same IEDB server using B-cell epitopes. Binding to both MHC-I and MHC-II alleles was predicted using a reference set of alleles available at IEDB [54,55]. Low percentile epitopes are regarded as high affinity binders (Baldauf et al. 2015). Then, the binding affinity of the considered epitopes was predicted through a server MHCPred 2.0, and those fulfilling the criteria of the selection (IC50 values < 100 nM for DRB*0101) were chosen. DRB*0101 is an allele that is responsible for HLA susceptibility and is found in 95% of the population [55] The selected epitopes were further processed in order to check their virulence through VirulentPred [56]. The antigenicity of the virulent epitopes was again checked through VaxiJen 2.0. The AllerTop server was then used for the removal of the allergic epitopes [57].

### 2.4. Design of a Multi-Epitopes Vaccine

One of the main issues concerned with epitope vaccines is that they are weakly immunogenic, but this can be solved by joining multiple antigenic epitopes through linkers and designing a multi-epitopes vaccine [58]. Multi-epitopes vaccines are considered a better option to combat pathogenic infections [59]. The linkers used for fusing the epitopes were GPGPG [59]. The final construct was joined to the cholera toxin [60]. The tool Protparam [61] was then used for predicting the physicochemical properties of the vaccine. The 3D structure of the vaccine was predicted through 3D pro [62], and then loops of the vaccine were modeled using Galaxy loop [63], and refinement was conducted via Galaxy refinement [64]. Disulfide bonds were introduced via design 2.0 [65] to achieve stability of the final construct. Codon optimization and reverse translation of the vaccine candidate were achieved through the Jcat tool [66].

### 2.5. Simulating Host Immunity against Vaccine

Using C-immSim server, host immune responses were simulated against the designed vaccine candidate [67]. This server predicted the host immune system simulation in response to the vaccine in three different organs, namely the thymus, bone marrow, and the lymph nodes [68].

### 2.6. Docking and Refinement

To understand the binding affinity of the vaccine with the receptors of the immune system, a molecular docking study was performed [69]. This analysis is very crucial because the high affinity of the construct with the receptors means that the construct could generate good immune responses [70]. The vaccine construct was blindly docked to the MHC-I, MHC-II, and Toll-like Receptor 4 (TLR-4) [71]. TLR-4 helps in the production of cytokines, which leads to the activation of adaptive immunity. Molecular docking was carried out through a patchdock tool [72]. After docking, refinement of the docked complexes was carried out via Firedock [73]. After Firedock, only the complex with lowest energy was considered [73]. The intermolecular interactions of the complexes were interpreted through Chimera 1.13.1 [74].

### 2.7. Molecular Dynamic Analysis

The vaccine was evaluated in 300ns of a computer simulation. The purpose of this step was to decode the dynamics affinity of the vaccine construct for the immune receptors used. Furthermore, it helps to examine the epitopes presentation of the vaccine to the host immune system [75]. AMBER 20 was used for simulating the docked vaccine complexes [76]. In the first phase, an antechamber program was used to generate the complexes’ parameters [77]. The complexes were then solvated in a TIP3P solvation box (size 12 Å) [78] via the Leap program [79]. For the illustration of the intermolecular interactions of the systems, a ff14SB force field was applied [80]. Nine, eight, and seven Na+ counter ions were added for neutralization of the TLR4, MHC-I, and MHC-II complexes, respectively. The second phase was the preprocessing phase, which was about preparing the systems to be used in the production. The energy of the systems was optimized using the steepest descent and conjugate gradient algorithms. Systems were heated up to 300 K. Langavin dynamics [81] was used for a constant temperature, while a SHAKE algorithm was used to constrain the systems’ hydrogen bonds [82]. Moving further, the complexes were equilibrated for 100-ps. Pressure equilibration was conducted via an NPT ensemble [83]. Simulation trajectories of 300-ns were generated using the Berendsen algorithm [84]. A SHAKE algorithm was used to constrain hydrogen bonds, and trajectories analysis was performed through CPPTRAJ [85].

### 2.8. Calculation of TLR4-Vaccine Binding Energies

The Molecular Mechanics Poisson-Boltzmann Surface Area (MMPBSA) binding free energies for the vaccine-TLR4 were estimated through an AMBER20 MMPBSA.py module [86]. The Ante-MMPBSA.py module of AMBER was used for the prediction of the input parameter files of the complex, receptor, and vaccine. The binding energies of 100 different frames were determined, and the purpose was to determine the difference of free energy between the solvated and gas phases [87].

## 3. Results

A number of *A. baumannii* genome sequences are available in public databases, which can be retrieved for experimental and computational research works. In this study, we selected 50 complete sequenced *A. baumannii* genomes that consist of approximately 95,050 proteins.

### 3.1. Bacterial Pan-Genome Analysis

**Figure 2:**
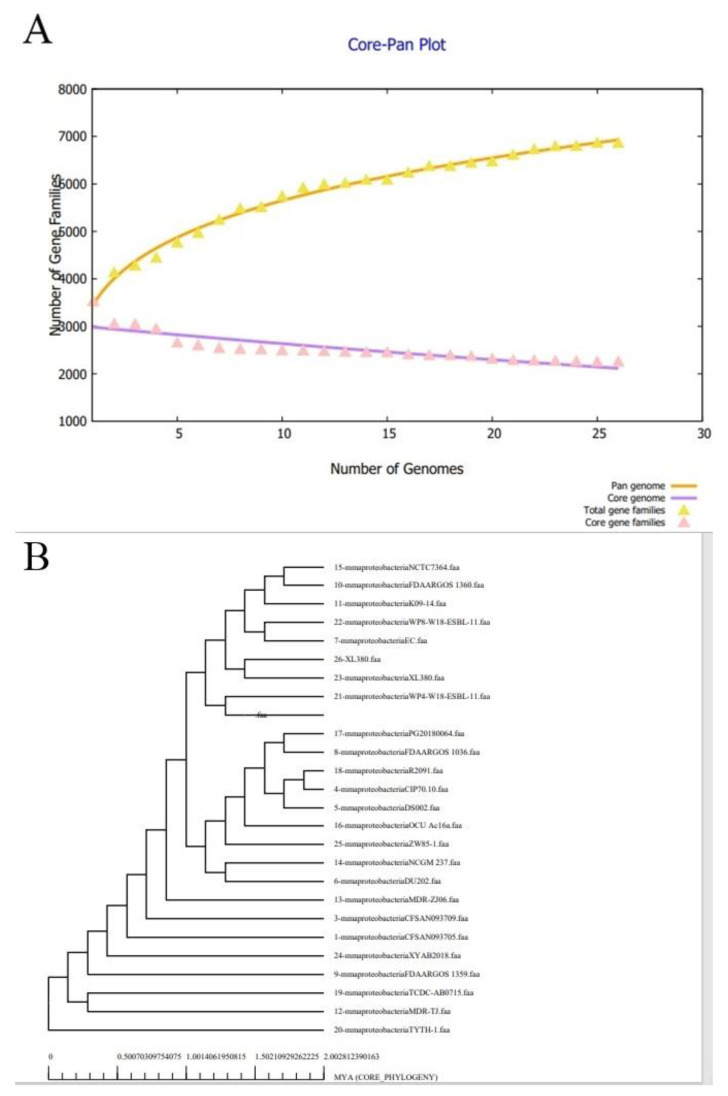
(**A**). pan- core plot (**B**). Core phylogeny tree of 50 complete genome of *A. baummanni*.

The pan-genome is the set of all genes present in a genome and contains a core genome (present in all strains), an accessory genome (those sequences that are present in two or more than two strains but not in all strains), and unique sequences (present only in a single strain) [88,89]. Bacterial pan-genome analysis was performed to obtain insights about the pathogen core genome. The *A. baummanni* strains have 95,050 core proteins (average number is 1901 proteins per genome). Accessory, unique, and exclusively absent proteins numbered 15,200, and 0, respectively [87]. The total number of proteins of each strain is graphically presented in Figure 2. The core-pan plot indicates that the strain’s pan-genome is in an open state, and chances are high of it gaining new genes in the future. Additionally, COG distribution found the core proteins involved in metabolic biogenesis [90].

### 3.2. CD HIT and PSORTB Analysis

**Figure 3:**
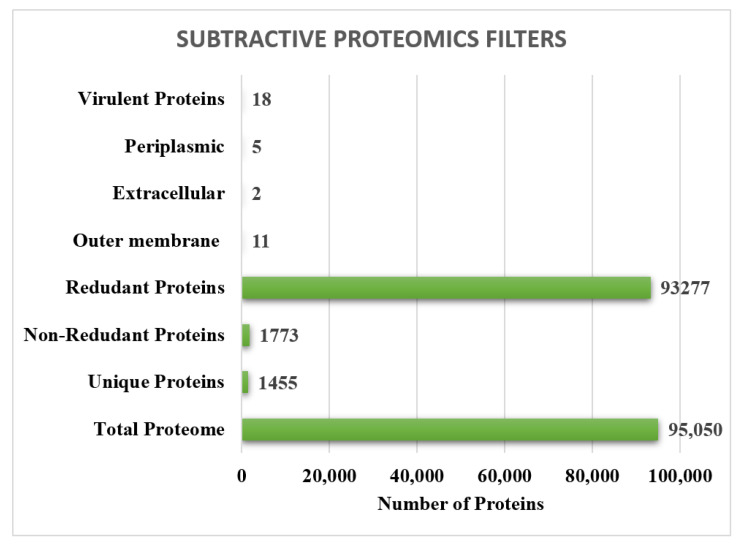
Total, unique, redundant, non-redundant, outer membrane, extracellular and periplasmic membrane, and virulence factors obtained through subtractive proteomics.

The Cd-Hit is a server used for the identification of redundant and non-redundant sequences in a complete genome. Cd-hit analysis of the core sequences was performed to remove the redundant sequences. The redundant sequences were removed, and the non-redundant sequences were selected for further processing. CD hit analysis showed 1773 non-redundant proteins in the pathogen core proteome and were processed further. The number of redundant and non-redundant proteins is shown in Figure 3. Those proteins that are present on the surface of pathogen were considered as good vaccine candidates because they are exposed to the environment and have the potential to produce an immune response in the host. Therefore, proteins that are present in the extracellular, outer membrane, and periplasmic space are considered to be usable for vaccine designing. After PsortB analysis, 2 extracellular-, 11 outer membrane-, and 5 periplasmic proteins were unveiled. The localization of proteins is explained in Figure 3.

### 3.3. Antigenicity, Allergenicity, Human and Normal Microbiota Similarity, and Transmembrane Helices and Stability Analysis

**Figure 4:**
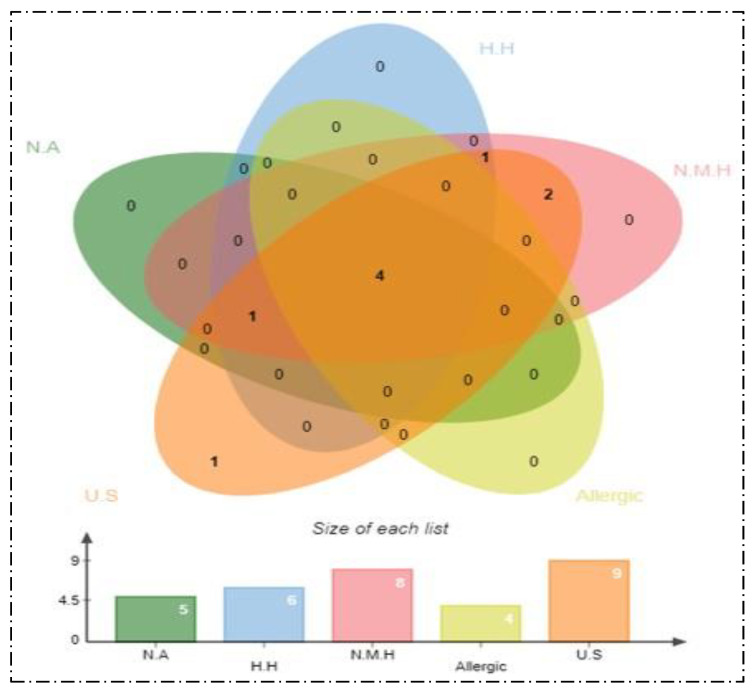
Number of non-antigenic (NA), human homologous (H.H), normal microbiota homologous (N.M.H), allergic and un-stable (U.S) proteins.

All 18 filtered proteins were checked for antigenicity, allergenecity, and homology with human and normal flora genomes through Vaxijen, Allertop, and BLASTp, respectively. Antigenicity analysis predicted 13 proteins as antigenic with a score > 0.5. The Allertop 2.0 server found 4 protein sequences as allergic. Six proteins were homologous to the human genome, and 8 proteins were similar to three normal microbiota species. The non-homologous proteins reduce the chances of stimulating auto-immune reactions [91]. Likewise, no hits against probiotic bacteria demonstrated that beneficial microorganism growth will not be altered. Furthermore, transmembrane helices analysis revealed 0 proteins. Nine proteins were discarded as unstable (score > 40) because they had a molecular weight of >100 kDa (Figure 4). Out of 18 proteins, only 4 proteins were antigenic, non-allergen, and non-homologous. These proteins are: TonB-dependent siderophore receptor, two OmpA family proteins, and OprD family outer membrane porin; these were selected as the best vaccine candidates.

### 3.4. Physiochemical Properties

Different physicochemical properties of proteins can be predicted through protparam. The most important is molecular weight. Low molecular weight proteins (less than 110 kDa) have only such proteins as can be purified easily. The physiochemical properties of 18 virulence factor molecules are shown in Table 1.

### 3.5. Prediction of B Cell Epitopes

After performing the subtractive proteomic filters, 4 proteins were prioritized. B-cell and T-cell epitopes were predicted for all the selected vaccine candidates. The binding of antigen and antibody is necessary for making an antigen-antibody complex to stimulate the immune system. The adaptive immunity is specific in pathogen clearance [92]. Adaptive immunity converts B-cells into plasma cells that generate antibodies that recognize the pathogen on succeeding encounters [93]. These immunological responses are key in vaccination [94]. The four selected proteins—TonB-dependent siderophore receptor, two OmpA family protein, and OprD family outer membrane porin—were subjected to B-cell epitopes prediction. B-cell epitopes predicted for the mentioned proteins are in the following order: 15, 5, 4, and 11, respectively, as tabulated in Table S1. The B and T lymphocyte cells of the acquired immune cells are involved in provoking antibodies-dependent responses against invader microorganisms [95]. Thus, in this study, the final predicted B-cell epitopes were subjected to T-cell epitopes prediction. The selection of MHC-I and MHC-II epitopes [96] is based on the least percentile score as shown in Table S2. The human leukocyte antigen (HLA) system (MHC in humans) is a vital part of the human immune system. The HLA system is controlled by genes present on chromosome 6 and encode cell surface molecules that present antigenic peptides to the T-cell receptor (TCR) on T cells [97].

### 3.6. Epitope Filtration Phase

In the epitope filtration phase, predicted epitopes were subjected to evaluation of different checks, mainly including HLA DRB*0101 allele binding efficacy and antigenicity analysis. The HLA DRB*0101 gene is highly prevalent in the human population (95%) [98]. Epitopes of IC50 values < 100 nM for DRB*0101 were selected as they represent efficient immune responses stimulating epitopes (Table 2). The epitope filtration phase revealed all the predicted epitopes as probably antigenic, non-allergic, non-toxic, and water soluble [99], as shown in Table 2.

### 3.7. Multi-Epitopes Vaccine Construct Designing

**Figure 5:**
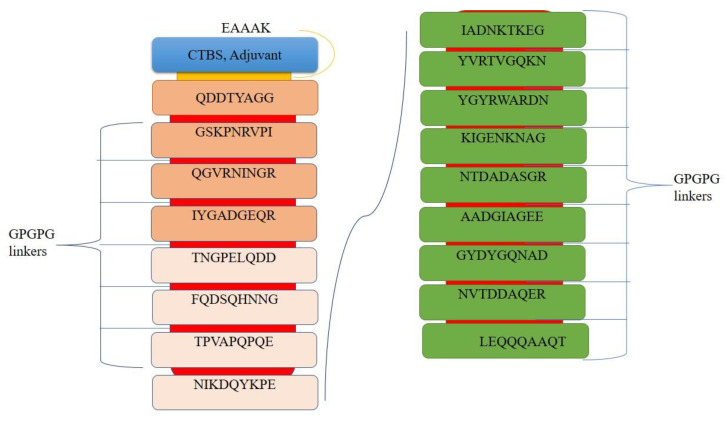
Schematic diagram of 250 amino acids long vaccine construct.

The issue with epitope vaccines is that they are weakly immunogenic to induce an immune response, which can be resolved by joining the epitopes to make a multi-epitopes vaccine [100,101,102]. Adjutants and linkers are used to make a multi-epitopes vaccine. In total, 17 epitopes were selected based on their ability to clear all vaccine filters and were joined together by using GPGPG linkers. These linkers keep the epitopes separate and do not allow them to fold around each other. The designed vaccine construct is shown in Figure 5. The designed vaccine stability score and half-life is 33 and 10 h in *E. coli*, respectively.

### 3.8. 3D Structure Prediction

**Figure 6:**
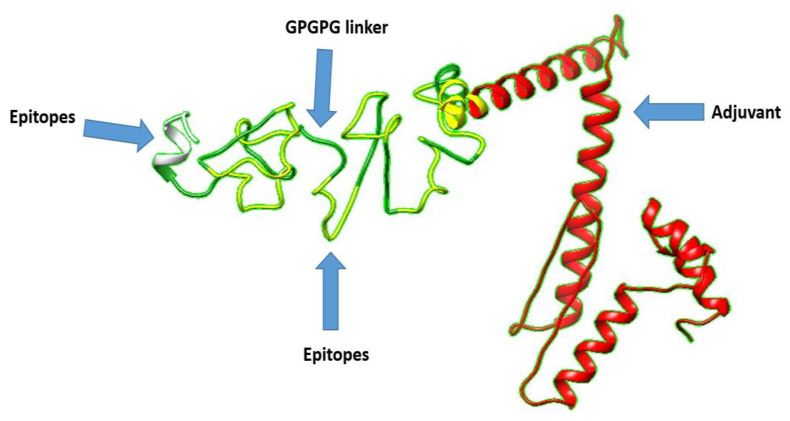
3D structure of vaccine construct. Red color adjuvant, forest green color show GPGPG linkers while yellow color represents epitopes.

The construct was checked for stability, and the result showed that the designed vaccine construct is stable and can be used experimentally because of its small size. Subsequently, a 3D structure was predicted and is given in Figure 6.

### 3.9. Loops Modeling and Refinement

The stability of the vaccine construct is very important, and to increase the stability, loops modeling was performed to design an efficient 3D vaccine. Residues involved in loop formation, including, Met1-Val8, Ala19-Gly21, Cys30-Thr36, Glu50-Ile61, Thr62-Pro74, Gly185-Ile186, gly199-Gln200-Lys207, Arg202-Pro220, Gln221-Gly227, Gly228-Pro240, and Ser240-Phe220, were selected for loop modeling.

### 3.10. Disulfide Engineering

**Figure 7:**
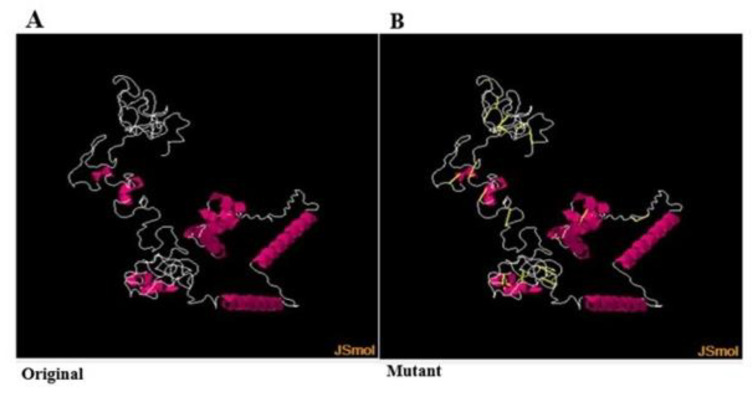
Original vaccine structure (**A**) and muted vaccine structure (**B**). The yellow stick in the mutated vaccine represents disulfide bonds.

Disulfide bonds provide substantial stability to the protein. Disulfide engineering incorporates new disulfide bonds to replace highly unstable pair residues. Disulfide engineering was performed to further increase the stability of the folded vaccine construct by decreasing the conformational entropy. The intra and intra chains of the vaccine were checked for disulfide. Both original and mutant structures are shown in Figure 7. The following residue pairs were found to be highly unstable and were mutated. These residues are: Ala20Cys -Tyr25Cys, Cys27-Cys84, Thr36Cys-Cys95, His48Cys-Ser80Cys. Cys85-Cys110, Cys65-Gln69Cys, Cys65-Cys72, Ser90Cys -Ala96Cys, Lys104Cys -Ser123 Cys, Ile107Cys -Asp121Cys, and Val108Cys -Pro117Cys.

### 3.11. Codon Optimization

Codon optimization refers to a genetic approach to optimizing a given sequence as per the translation machinery of the host to obtain maximum expression of that exogenous sequence in the host expression system [103]. The codon optimization of the vaccine,, which is 0.92, was measured by a codon adaptation index (CAI); the GC content of the vaccine is 57.08%. These values indicate the efficient codon usage of the vaccine sequence in the *E. coli* K12 strain and hence it greater expression. 

### 3.12. Molecular Docking

The interaction of the designed vaccine with the host receptors is necessary to stimulate the immune response against the designed vaccine. Blind docking of the vaccine was carried out with MHC-I, MHC-II, and TLR-4 receptors. The immune cell receptors were retrieved from an NCBI database using their specific PDB ID. The results of the blind docking are given in Tables S3–S5. In each case, 20 solutions were predicted, mainly the docking score.

### 3.13. Refinement of Docked Complexes

**Figure 8:**
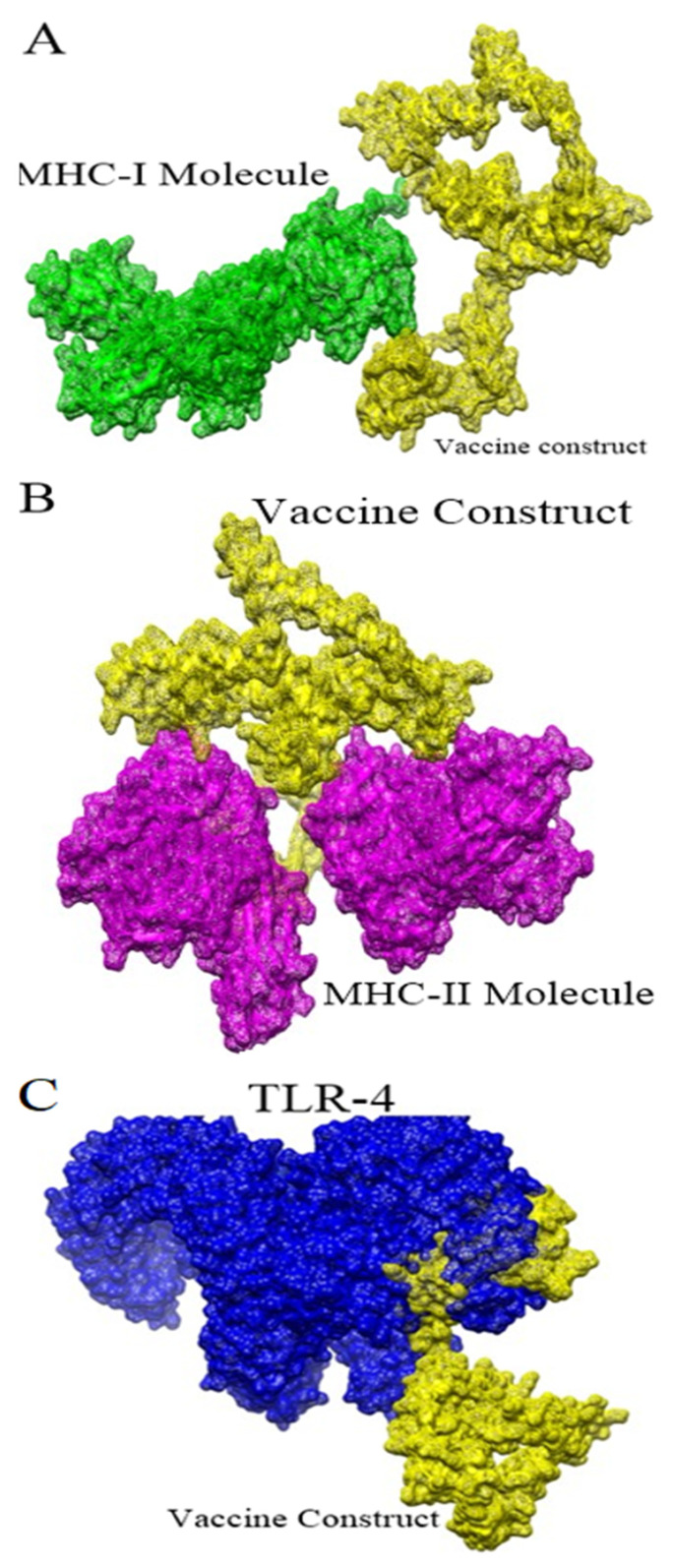
Docked intermolecular conformation of the vaccine to (**A**) MHC-I, (**B**) MHC-II, and (**C**) TLR-4.

From the blind docking, the top 10 complexes were selected for refinement [59]. The top 10 solutions for each receptor are tabulated in Table 3, Table 4 and Table 5. Low global energy solutions were selected for binding conformation analysis [60]. The binding mode and interactions of the vaccine with TLR4, MHC-I, and MHC-II were studied. The selection of the top complex was carried out based on global binding energy. In the case of MHC-I, solution 7 was selected considering its global energy of −17.35 kcal/mol. For MHC-II, complex 8 was selected considering its global binding energy of −2.99 kcal/mol. For TLR-4, solution 2 was selected with a global binding energy of −3.22 kcal/mol. The docked intermolecular conformation of the vaccine to MHC-I, MHC-II, and TLR-4 is shown in Figure 8. It was also noticed that among the shortlisted solutions for each immune receptor were found different intermolecular conformations.

### 3.14. Chemical Interactions of the Vaccine with MHC-I, MHC-II, and TLR-4

Antigen presentation by MHC proteins is essential for acquired immunity. Prior to presentation, peptides must interact with various types of immune cells. These intermolecular interactions are critical to decipher, as they highlight the residues important from a vaccine recognition perspective. The designed vaccine showed robust interactions with several key hydrophilic and hydrophobic residues of receptor molecules, which are mentioned in Table 6.

### 3.15. Molecular Dynamic Simulation

**Figure 9:**
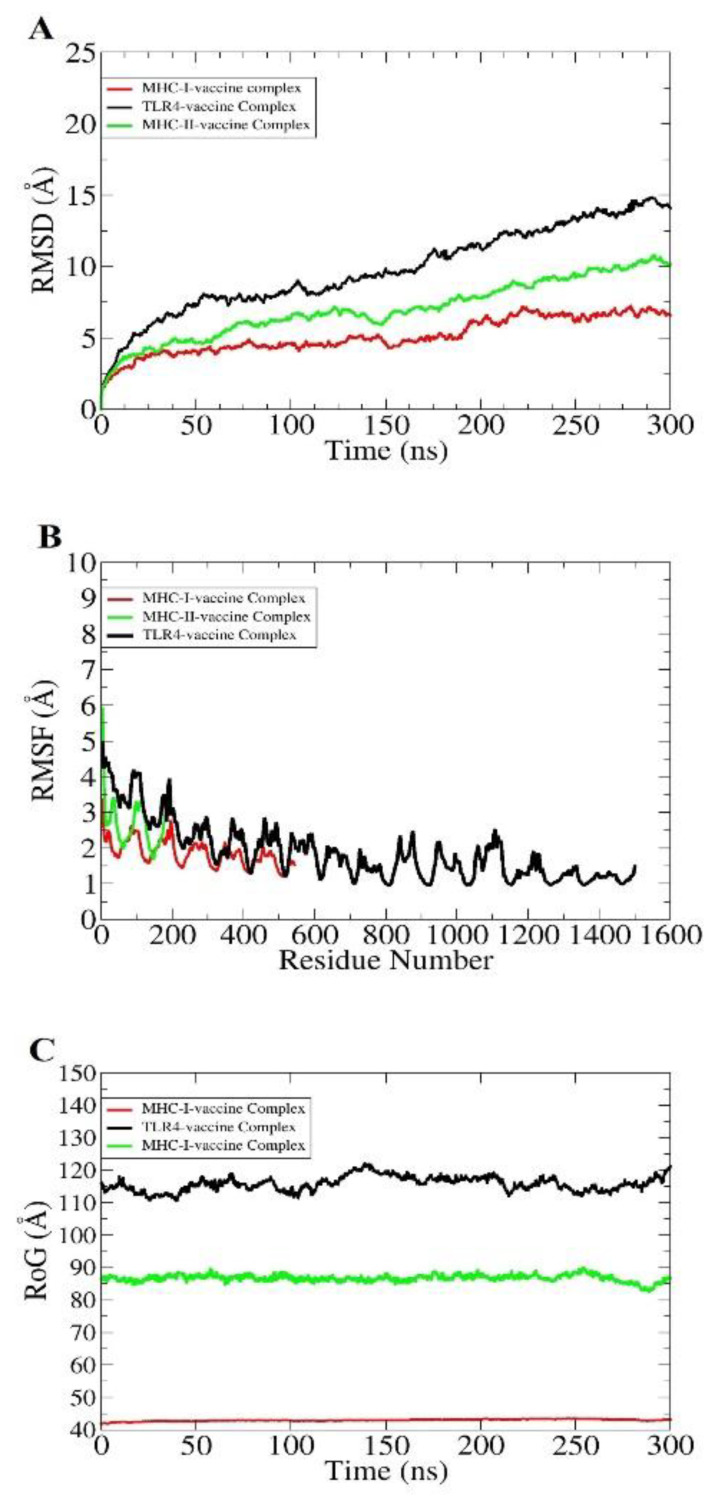
Statistical analysis of the molecular dynamics simulation trajectories. RMSD (**A**), RMSF (**B**), and RoG (**C**).

The dynamics of the docked complexes were deciphered in order to make sure whether the binding of the vaccine to receptors was stable and whether the epitopes were in an exposed position to the host immune cells. The dynamics stability was evaluated through (i) root mean square deviation (RMSD), (ii) root mean square fluctuation (RMSF), and the (iii) radius of gyration (RoG) [104]. All these three types of analysis were conducted considering carbon alpha atoms. The RMSD plot of the systems was seen to steadily increase with no major deviations seen. This implicates the stable intermolecular binding between vaccine and receptors throughout the length of the simulation time. This also confers the continuous presentation of vaccine epitopes to the immune system for activation of humoral and cellular immunity. The maximum RMSD for each system reaches 10 Å (Figure 9A). The higher RMSD is the result of a large vaccine-receptors complex, but despite that, the vaccine is strongly held at the docked site of the receptors. Second, the RMSF was calculated to determine the residue level fluctuations of receptor molecules (Figure 9B). This was vital, as the presence of vaccine either heightens the residues; stability or makes it highly unstable. Most of the receptor residues show good stability (<3 Å). The loops are flexible, and in the presence of the vaccine, such loops become highly flexible, showing a high RMSF. Lastly, RoG assay was conducted to examine the complexes’ compactness with respect to time (Figure 9C). The RoG complemented the RMSD and concluded that the systems are in an equilibrium state.

### 3.16. Calculation of Vaccine-Receptors Binding Energies

Binding free energies of the docked molecules were estimated through MM-GBSA, and MM-PBSA techniques were used for validation of the binding efficacy of the docked complexes. The total binding free energies of the MHC-I, MHC-II, and TLR-4-vaccine complex were −178.62 kcal/mol, −181.94 kcal/mol, and −169.83 kcal/mol, respectively (Table 7). A major contributor to the binding net energy came from the electrostatic energy as well as van der Waals energy, and a non-favorable (not contributing to the overall stability of the complexes) contribution was seen from solvation energy.

### 3.17. Immune Simulation of the Designed Vaccine

**Figure 10:**
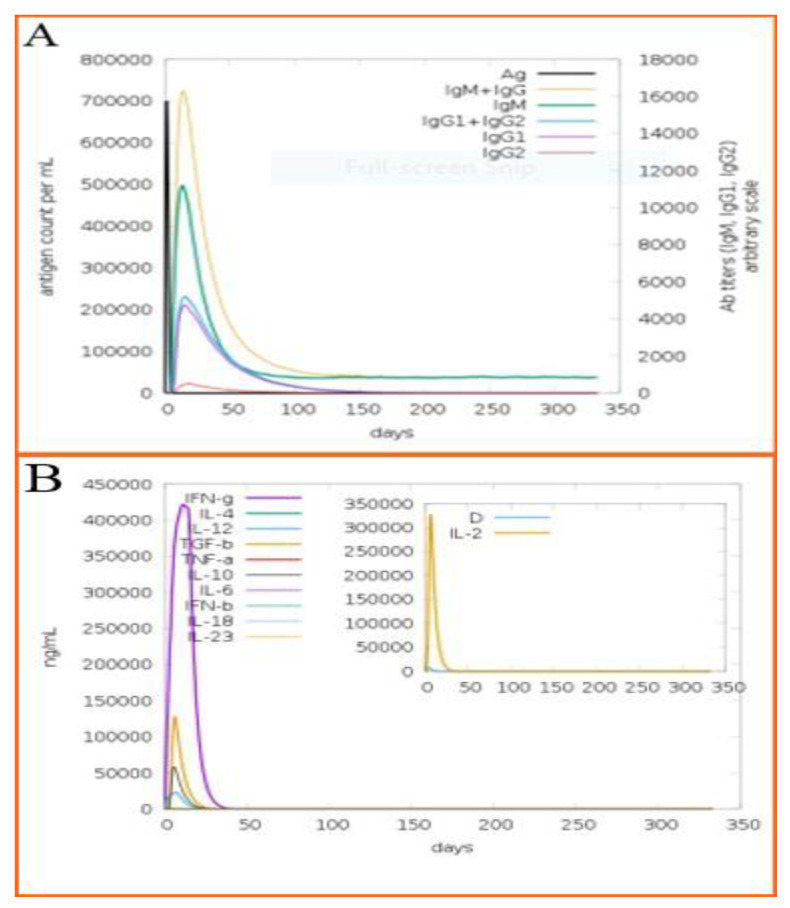
(**A**) Immunoglobulin titer as shown through different color peaks in response to multi -epitopes vaccine injection as shown with black color peak (**B**) Elicitation of interleukins level after injection of multi-epitopes vaccine construct as represented by C- immune simulation analysis.

In this analysis, the host response against the modeled vaccine was predicted for 350 days and it was analyzed that there is a high production of IgG and IgM antibodies against the designed vaccine. The secondary response was followed by a tertiary immune response, which led to the high-level production of B-cells and “IgM + IgG, IgM, IgG1 + IgG2, IgG1 and IgG2” (Figure 10A). An increase in interferon was also detected for 350 days, and the results are mentioned in Figure 10B. The different B-cell and T-cell immune responses in response to the antigen are given in Figures S1 and S2.

## 4. Discussion

We are moving towards the end of the antibiotic era. The crisis of AR and new bacterial strains are emerging. Some bacterial species have already been declared as resistant to all available antibiotics [105]. *A. baumannii* is one of those species of bacteria that has been reported as resistant to all available antibiotics [106]. *A. baumannii* is also capable of adopting new mechanisms of resistance and is evolving very quickly [107]. *A. baumannii*, is also able to develop a quick resistance to antibiotics due to its plastic genome and has the capability of causing healthcare associated infections and has the potential to survive in highly detrimental conditions [108]. The growing burden of microbial resistance has a remarkable clinical and economical effect and demands the development of new therapeutic candidates to treat infections of bacterial pathogens. 

Vaccines have the potential to provide a permanent solution to tackling AR bacteria. RV, which is a genome-based vaccine development technique, is a popular approach in identifying new vaccine candidates [109]. RV was developed by Dr. Rino Rappuoli and is an emerging in silico vaccine development framework [109,110]. In the recent past, RV has contributed significantly to vaccine development against pathogens that are unable to be addressed by Pasteur’s principles of vaccinology [17]. The traditional Pasteur vaccinology approach is not an appropriate choice for pathogens that cannot be cultured [111]. Likewise, it ignores conservation and molecular mimicry of antigenic determinants [79], as surfaced in vaccine development against *Neisseria* and *Mycobacterium* species [112]. The choice of subunit vaccines is also very costly and time consuming, and the likelihood of screening potential antigenic epitopes is low [113]. The availability of an exponential amount of genomic data in public databases and advancement in bioinformatics tools have significantly sped up the vaccine development process [114]. Vaccine candidates can be identified at the pathogen surface through RV [112], which has been successfully used in meningococci serogroup B vaccine (4CMenB) development [111]. RV has been extensively exploited for designing vaccines against many bacterial and viral pathogens [35]. Lai et al. used glycoprotein from *Ebolavirus* and designed an MHC-I epitope-based vaccine by employing an RV approach and subsequently obtained promising results for a proposed vaccine via experimental validation (https://doi.org/10.3389/fmicb.2017.01571 accessed on 22 April 2022). In another study, Agallou et al. performed mice experiments of eight MHC-I and MHC-II epitopes predicted using an RV approach and proposed that these epitopes can provide better immunity against *Leishmania infantum* (https://doi.org/10.3389/fimmu.2014.00268 accessed on 22 April 2022). Additionally, Singh et al. predicted FilF as a potential vaccine target using RV and immunoinformatics approaches and validated its 50% immune protective efficacy in a murine pneumonia model (https://doi:10.3389/fmicb.2016.00158 accessed on 22 April 2022). In recent advancements, classical reverse vaccinology is integrated with pan-genomic-based reverse vaccinology (PGRV) to identify core genome antigens. PGRV is successfully used to map four protective antigens in *Streptococcus agalactiae* genomes [114,115]. There is no vaccine in the development process for *A. baumannii*, which makes the situation more worrisome. Therefore, substantial efforts were needed to identify protective antigens from the core genome of the pathogen, which can easily be analyzed in experimental studies.

The current study was about computational prioritization of vaccine candidates against *A. baumannii* based on RV and pan-genome analysis. In the current study, we report four outer membrane proteins: TonB-dependent siderphore receptor, OmpA family protein, type IV pilus biogenesis stability protein, and OprD family outer membrane porin, all of which highly susceptible to an effective vaccine. The OmpA is a well conserved protein among *Acinetobacter* species and can produce broad-range immunity. Besides this, OmpA has a soluble and stable structure, making it an attractive vaccine target. This protein also has favorable adhesion and haemagglutinins properties. However, as the OmpA pure form is insoluble, its delivery is hard (https://doi.org/10.22038/ijbms.2019.30799.7427 accessed on 22 April 2022). The combination of OmpA antigenic epitopes with those of the TonB-dependent siderphore receptor, type IV pilus biogenesis stability protein, and OprD family outer membrane porin might generate an ideal vaccine construct capable of generating strong and protective immune responses. The findings of the study will save time and may lead to cost effective vaccine development. We used RV in combination with immunoinformatics and biophysical approaches to construct a chimeric vaccine against superbug *A. baumannii* and understand its binding potential with the host MHC-I, MHC-II, and TLR-4 receptors to examine its immune system presentation and ability to confer protection against the antigen. At the end, a host immune system simulation was performed against the vaccine in order to predict the antibodies’ production and cellular immune responses. This in silico simulation has many limitations, for instance, it does not include type I IFN, which has been recently evident as potentially important in the management of infection and can be further improved using more experimental knowledge. To date, many attempts have been made to develop vaccines against *A. baumannii*, yet there is no successful vaccine against this pathogen that has been identified [116,117,118]. Several attempts were also made to form a live attenuated vaccine against *A. baumannii*, and recently a D-glutamine strain of pathogen was used to make a live attenuated vaccine. A strain of mutated morl1 and morl2 genes was synthesized. Although it induced both antibody and cell mediated immunity, the genes were less virulent and the response was weak [119].

## 5. Concluding Remarks and Limitations

In this in silico study, several different bioinformatics techniques, ranging from subtractive proteomics to immunoinformatics techniques, were considered, with the aim of designing a multi-epitopes-based vaccine against a nosocomial superbug *A. baumannii* bacteria. These techniques have been applied to design vaccines against different pathogens [116,117,118]. The rationale behind this work is the lack of an FDA approved vaccine for *A. baumannii*, and the pathogen shows a high level of resistance to many available antibiotics [120]. The designed vaccine construct comprises only antigenic epitopes that do not lack allergic sequences, but it is capable of eliciting strong immune responses. The epitopes were filtered from four vaccine proteins prioritized using vaccine candidacy criteria. These proteins are: TonB-dependent siderphore receptor, OmpA family protein, type IV pilus biogenesis stability protein, and OprD family outer membrane porin. The designed vaccine construct has shown excellent binding potency to the immune cell’s receptors, disclosed proper binding confirmation, and produced strong binding energies. We believe that the findings and predictions of our study may accelerate the vaccine development process against *A. baumannii* and may deliver data to speed up vaccine development against the pathogen. Moreover, the findings of the study will also save time and cost and will be helpful for vaccinologists in developing a vaccine against *A. baumannii*. Although we were very careful in the selection process of epitopes, the study has some limitations. The vaccine construct epitopes’ proper fusion needs thorough experimental testing, which is not conducted herein. The predictions made in the study are based on tools and servers. These predictions are not that accurate due to the lack of healthy experimental data to train the tools and servers used. Lastly, the real immune protection of the designed vaccine required a wide range of in vivo and in vitro testing studies.

## Figures and Tables

**Table 1:** Physiochemical properties of the selected virulence factor molecules including shortlisted four potential vaccine candidates.

Outer Membrane Proteins	Encoded Proteins	Amino Acid	Molecular Weight	Gravy	Aliphatic Index	Instability Index	Theoretical (PI)
>core/109/1/Org1_Gene719	Type II toxin-antitoxin system antitoxin maze3	1071	115.65	−0.336	87.06	30.04	9.35
>core/298/1/Org1_Gene3311	Methionine synthase [Mycobacterium tuberculosis	775	84.63	−0.391	76.68	28.67	8.58
>core/315/1/Org1_Gene2327	Hypothetical protein	758	80.95	−0.222	96.37	30.48	8.86
>core/982/1/Org1_Gene2259	MULTISPECIES: 1-acyl-sn-glycerol-3-phosphate acyltransferase	484	52.26	−0.311	92.02	39.59	9.02
>core/1058/1/Org1_Gene66	Nuclear transport factor 2 family protein [Mycobacterium tuberculosis	471	48.39	−0.015	100.81	32.8	5.59
>core/2148/1/Org1_Gene1404	Hypothetical protein K60_024290 [Mycobacterium tuberculosis variant bovis BCG str. Korea 1168P	356	38.97	−0.396	80.93	32	5.32
>core/3446/1/Org1_Gene2677	Chromosome segregation protein SMC	266	30.44	−0.415	85.23	41.44	9.01
>core/4300/1/Org1_Gene2340	NlpC/P60 family peptidoglycan endopeptidase RipB	217	22.13	−0.288	84.61	30.23	9.3
>core/1212/7/Org7_Gene3542	MULTISPECIES: zf-HC2 domain-containing protein [Mycobacterium tuberculosis complex	451	49.38	−0.42	70.35	21	5.66
>core/3840/27/Org27_Gene62	MULTISPECIES: hypothetical protein	244	26.05	−0.347	80.82	42.43	6.44
>core/4074/16/Org16_Gene452	Ribosomal protein S7 from Mycobacterium tuberculosis	211	25.4	0.08	112.81	31.57	5.74
**Extracellular Proteins**							
>core/331/1/Org1_Gene3212	DUF427 domain-containing protein	742	83.32	−0.556	69.46	35.45	6.15
>core/5806/1/Org1_Gene1744	D-alanyl-D-alanine carboxypeptidase	143	15.5	-0.09	84.06	25.08	5.52
**Periplasmic Proteins**							
>core/3114/1/Org1_Gene3080	D-alanyl-D-alanine carboxypeptidase dacb2	288	31.25	−0.318	95.14	19.93	5.93
>core/466/2/Org2_Gene2561	Glycine cleavage system aminomethyltransferase gcvt	661	69.36	−0.241	78.11	35.08	5.46
>core/1121/3/Org3_Gene1840	MULTISPECIES: multidrug efflux SMR transporter Mmr	463	49.7	−0.114	96.2	35.66	9.34
>core/2579/16/Org16_Gene524	Conserved protein of uncharacterised function	324	36.57	−0.223	92.96	30.15	5.81
>core/4275/27/Org27_Gene2284	Site-2 protease family protein [Mycobacterium tuberculosis	219	24.1	−0.401	76.8	21.59	5.88

**Table 2:** List of potent DRB*0101 binders, probable antigenic, non-allergic, non-toxic, and good water-soluble epitopes.

Epitopes	DRB*0101 Binder Score	Antigenicity	Allergenicity	Water Solubility	Toxicity
LEQQQAAQT	7.635	Probable antigenic	Non-Allergic	Good water soluble	Non-toxic
QDDTYAGGQ	12.74				
GSKPNRVPI	13.55				
QGVRNINGR	17.02				
IYGADGEQR	63.53				
TNGPELQDD	41.78				
FQDSQHNNG	5.85				
TPVAPQPQE	52.97				
NIKDQYKPE	22.86				
IADNKTKEG	77.62				
YVRTVGQKN	47.32				
YGYRWARDN	6.41				
KIGENKNAG	83.18				
NTDADASGR	3.29				
AADGIAGEE	36.73				
GYDYGQNAD	27.42				
NVTDDAQER	1.17				

**Table 3:** Top 10 refined docked complexes of vaccine to MHC-1 and model vaccine construct generated by FireDock. ACE (Atomic contact energy) and HB (Hydrogen bonding).

Rank	Solution Number	Global Energy	Attractive VdW	Repulsive VdW	ACE	HB
1	7	−17.35	−6.20	−1.65	−4.99	−1.25
2	5	25.32	−33.83	17.69	13.88	−0.26
3	9	436.06	−52.43	675.26	−3.37	−6.49
4	1	1559.57	−83.37	2066.11	15.99	−8.89
5	6	2178.22	−50.99	2740.59	29.10	−11.89
6	10	4719.15	−72.39	6014.70	16.31	−8.98
7	8	6838.22	−94.58	8700.09	6.24	−14.44
8	3	13,952.77	−95.41	17,578.41	16.24	−17.85
9	4	13,994.61	−146.04	17,861.02	−19.01	−27.64
10	2	22,692.17	−198.31	28,778.51	3.39	−32.98

**Table 4:** Top 10 refined docked complexes of vaccine to MHC-II and model vaccine construct generated by FireDock.

Rank	Solution Number	Global Energy	Attractive VdW	Repulsive VdW	ACE	HB
1	8	−2.99	−4.29	1.49	4.17	−1.27
2	7	7.20	−1.75	0.00	1.92	0.00
3	3	20.83	−4.65	5.59	1.20	−0.27
4	9	23.60	−5.65	0.78	4.63	−0.30
5	5	35.96	−25.25	36.41	14.29	−3.90
6	10	164.06	−15.38	199.72	13.40	−1.43
7	4	1426.58	−34.55	1827.26	1.63	−2.65
8	6	2708.43	−65.54	3541.68	−9.12	−2.25
9	1	7877.71	−87.43	9990.68	12.16	−9.37
10	2	10,558.78	−89.75	13,391.88	−3.44	−16.32

**Table 5:** Top 10 refined docked complexes of vaccine to TLR4 and model vaccine construct generated by FireDock.

Rank	Solution Number	Global Energy	Attractive VdW	Repulsive VdW	ACE	HB
1	2	−3.22	−26.77	7.43	21.39	−2.28
2	7	7.57	−2.84	0.00	1.28	0.00
3	9	10.66	−15.81	32.02	2.23	−1.76
4	10	28.75	−11.25	3.62	13.23	−0.50
5	6	29.86	−11.03	1.79	10.45	−1.72
6	1	915.94	−48.21	1190.12	17.81	−4.33
7	5	3386.99	−52.35	4311.65	8.99	−5.38
8	4	4737.61	−121.11	6190.84	−3.73	−16.92
9	3	7186.19	−93.88	9131.91	16.17	−11.81
10	8	7763.02	−85.23	9873.19	2.20	−7.86

**Table 6:** Residues vise interaction of docked complex with MHC-I, MHC-II, and TLR-4 receptors.

Vaccine Complex	Interactive Residues
MHC-I	Asn346, Asn347, Arg131, Glu58, Glu60, Glu161, Glu53, Ile 46, Lys127, Leu 126, Met138, Pro57, Ser132, Try135, Thr124, Leu64, Phe8, Try50, Try133
MHC-II	Ile187, Ser218, Ser240, Val203
TLR-4	Asn58, Asp84, Asn176, Asn173, Asn44, Cys 29, Glu42, Arg69, Asn112, Asp84, Arg67, Cys40, Cys148, Cys29, Tyr70, Leu17, Asp35, Thr92, Gln91, Glu136, Gln91, Glu31, Glu179, Glu143, Glu144, Glu111, Glu178, Glu31, His179, His8, Ile138, Tyr46, Glu89, Ile93, Lys108, Pro28, Pro113, Phe64, Phe147, Pro145, Pro142, Phe77, Pro78, Pro78, Leu74, Met40, Ser172, Ser141, Ser141, Ser126, Thr110, Leu85, Lys47, Lys30, Lys130, Tyr46, Leu66, Thr15, Leu152, Lys153, Ser105, Thr106, Lys57, Thr56, Thr37, Tyr38, Thr37, Val133, Thr37, Val35, Val30, Pro28, Val35, Val30.

**Table 7:** MM-GB/PBSA binding free energies calculation.

Energy Parameter	TLR-4-Vaccine Complex	MHC-I-Vaccine Complex	MHC-II-Vaccine Complex
**MM-GBSA**			
VDWAALS	−97.12	−106.08	−107
EEL	−105.68	−85.68	−88.07
EGB	48.01	32.39	28.24
ESURF	−15.04	−19.25	−15.11
Delta G gas	−202.8	−191.76	−195.07
Delta G solv	32.97	13.14	13.13
Delta Total	−169.83	−178.62	−181.94
**MM-PBSA**			
VDWAALS	−97.12	−98.36	−117.66
EEL	−105.68	−88.07	−54.05
EPB	43.25	23	28.28
ENPOLAR	−9	−14.19	−17.1
Delta G gas	−202.8	−191.76	−195.07
Delta G solv	34.25	8.81	11.18
Delta Total	−168.55	−182.95	−183.89

VDWAALS (van der Waals), EEL (electrostatic), Delta G gas (net gas phase energy), Delta G solv (net solvation energy), Delta Total (net energy of system).

## Data Availability

The data presented in this study are available within the article.

## Supplementary Materials

The following supporting information can be downloaded at: https://www.mdpi.com/article/10.3390/ijerph19095568/s1. Table S1. List of B cell epitopes predicted from four potential vaccine proteins. Table S2. MHC –I and MHC-II predicted epitopes. Table S3. Docking score of top 20 complexes of designed vaccine construct to MHC-I complexes generated by patchDock server. Table S4. Docking score of top 20 complexes of designed vaccine construct to MHC-II complexes generated by patchDock server. Table S5. Docking score of top 20 complexes of designed vaccine construct to TLR4 complexes generated by patchDock server. Figure S1. B cell and T cell population produced in response to the vaccine antigen. Figure S2. Immune cell response generated in response to chimeric vaccine construct. Tc (cytotoxic killer T-cell) Macrophages (Mφ) Nature killer cell, Dendritic and epithelial cell.
