# Supplementary material for: Vaccinomics to Design a Multi-Epitopes Vaccine for Acinetobacter baumannii
Source: Int J Environ Res Public Health. 2022 May 4;19(9):5568. doi: 10.3390/ijerph19095568 (PMC9104312; doi:10.3390/ijerph19095568)
Supplement: Supplementary file 1 [file ijerph-19-05568-s001.zip › ijerph-1636359-supplementary.pdf]

Table S1. List of B cell epitopes predicted from four potential vaccine proteins.

Protein Accession	Predicted Epitopes
**>core/298/1/Org1_Gene3311** (TonB-dependent siderphore receptor)	FVLNGVASAQIALRLGY
	SAKTLEQQQAAQTNVA
	QDDTYAGGQVSSKSSVGFLGNKT AMETPFNTIAYTEKYIADQQAKD
	IFSTGAYGVIA
	YMSDSQFGGHLDIGRRFGEN
	YRDGEGPIKEQEKT
	GVNLPKAPNPDTLLNPTWTVYNT
	KTEIQNEAGDI
	RETDDEAGIRQGFPEGDRVTN
	YNPNWGSKPNRVPIAPIFSNK
	VTESLNGQGVRNINGRYDKNA
	LSSGGTVPTGQNYENEGQIFSPF
	VIKSTDEKIKSIYGADGEQR
	TKTRIASELGNVVAG
	ASKQYIYNDNSTSVP
**>core/2148/1/Org1_Gene1404 (**OmpA family protein)	VLGGHLKPAAPVVEVAPV EPTPVAPQPQELTED
	FFDTNKSNIKDQYKPE
	NTGPRKLNERL
	FAWDQPIADNKTKEGRA
	RTVVVQPGQEAAAP
**>core/3446/1/Org1_Gene2677 (**type IV pilus biogenesis stability protein	EGSKPNLEKAEHY
	MERYNDAIE
	QQQIPAATQMYEQYVRTVGQKN
	RALFPESPEYQR
	LYGYRWARDNVGQ

**>core/1212/7/Org7_Gene3542 (**OprD family outer membrane porin)	EQSEAKGFVEDA
	DKKQGAKDTSS
	KIGENKNAGNQMIPKH NDGSAYDHWAR
	DQMSDQINTDADASGRGLD
	KQPLANDSS
	KFDANAHTYSATGTVAPNYAADGIA GEEK
	TGNVGYDYGQNADG
	DFGKLGVLPG
	WDIKVRNVTDDAQER
	SDAYQGAYIGDT

Table S2. MHC –I and MHC-II predicted epitopes.

MHC-1 (Epitopes)	Percentile Score	MHC-II (Epitopes)	Percentile Score
LEQQQAAQT	2.6	KTLEQQQAAQT	8
QDDTYAGGQV	18	QDDTYAGGQVSSKSS	1.80
GSKPNRVPI	0.31	YNPNWGSKPNRVPI	21
GQGVRNINGR	3.5	VTESLNGQGVRNIN	19
SIYGADGEQR	0.23	EKIKSIYGADGEQR	19
LTNGPELQDD	15	GNLTNGPELQDD	26
FQDSQHNNG	12	FQDSQHNNGGKDG	54
TPVAPQPQEL	0.07	PTPVAPQPQELTED	9.80
FDTNKSNIKDQYKPE	11	FDTNKSNIKDQYKPE	41
IADNKTKEGR	2.7	DQPIADNKTKEGRA	45
YVRTVGQKN	6.8	QMYEQYVRTVGQKN	8.40

YGYRWARDNV	14	YGYRWARDNVG	8.40
KIGENKNAG	18	KIGENKNAGNQMI	13
NTDADASGR	0.67	DQINTDADASGRGLD	36
AADGIAGEEK	1.3	VAPNYAADGIAGEEK	4.20
NVGYDYGQNA	1.2	GNVGYDYGQNADG	28
NVTDDAQER	0.17	WDIKVRNVTDDAQER	21

Table S3. Docking score of top 20 complexes of designed vaccine construct to MHC-I complexes generated by patchDock server.

**Solution No**	**Score**	**Area**	**ACE**	**Transformation**
1	22972	4577.9	385.16	-1.38 0.44 1.65 17.60 20.70 -25.44
2	21532	3064.3	215.95	-0.73 -0.32 -0.33 -38.41 -14.52 40.15
3	21376	4205.6	-26.00	2.44 1.15 -2.23 -26.96 -28.12 19.72
4	21348	4023.7	-183.75	2.71 1.24 -2.66 -29.08 -26.38 24.55
5	21084	3362.3	356.74	-0.10 -1.09 0.35 -1.56 38.52 -37.94
6	19992	3107.8	433.65	-1.88 0.16 3.06 17.09 -9.48 -1.29
7	19166	3697.1	29.85	3.06 -0.75 -1.90 -43.23 22.68 -84.90
8	18956	4994.1	121.34	-1.98 -0.73 0.18 -1.03 -3.71 1.30
9	18850	2397.2	432.49	-0.71 -0.26 1.53 -58.22 14.00 -64.53
10	18684	2465.1	83.37	0.19 -1.24 2.22 -10.41 -20.61 43.09
11	18326	3234.7	299.37	-2.93 -0.72 0.87 -33.55 39.11 -18.27
12	18274	2713.2	177.82	0.56 0.44 -2.89 -44.58 8.31 -90.58
13	18158	3342.3	119.17	0.81 -0.69 -1.19 -24.52 45.28 -53.00
14	18106	2987	457.09	-0.85 1.11 2.84 28.31 -6.30 -24.38
15	17954	2829.7	-434.35	2.59 -0.53 -0.90 2.18 42.68 -18.59
16	17952	3254.7	-28.62	-1.37 -0.47 -2.16 -8.31 -36.40 20.15
17	17726	2769.6	402.10	-1.41 -0.67 0.21 -10.45 -18.79 -72.53
18	17702	2283.6	168.14	-2.40 0.61 -1.82 7.23 20.55 -90.44
19	17674	3475.9	494.22	0.64 -0.33 3.13 -28.23 17.66 11.30
20	17422	3831	233.01	-0.55 0.73 1.63 -7.23 -2.77 -81.27

Table S4. Docking score of top 20 complexes of designed vaccine construct to MHC-II complexes generated by patchDock server.

Solution No	Score	Area	ACE	Transformation
1	20970	3793.4	98.40	0.93 0.46 -1.93 95.14 77.03 -13.40
2	20746	3719.3	256.36	-2.29 0.41 0.22 86.33 114.57 23.55
3	19600	3072.4	270.52	1.72 -0.29 -1.66 92.32 69.45 23.84
4	19124	3343	104.07	0.53 0.22 1.40 103.84 64.61 31.53
5	18786	2577.5	193.64	2.03 -0.63 1.36 131.68 78.92 11.70
6	18782	3442.8	234.48	-2.48 0.20 0.22 90.98 117.33 20.12
7	18616	3057.4	68.17	-1.86 -0.34 -2.27 87.76 84.53 21.70
8	18452	2755.8	430.87	-0.78 1.15 2.74 131.71 76.65 -0.46
9	18386	2278.3	286.41	-1.34 -0.51 -2.71 111.38 44.08 30.60
10	18348	3601.7	-29.78	-1.97 0.48 0.48 75.28 117.60 23.83
11	18276	3106.2	-55.80	1.00 0.45 -2.07 96.78 75.65 -15.48
12	18270	2688.6	142.73	0.58 0.32 -2.05 115.60 89.59 25.26
13	18066	2942.7	405.24	1.30 0.61 -0.12 97.68 78.64 29.76
14	18010	3149	188.61	1.81 1.04 3.05 130.21 67.10 26.46
15	17968	3233.4	174.35	-2.02 0.48 0.46 74.95 116.35 25.62
16	17754	2775.6	414.06	0.60 1.29 1.38 114.19 95.78 6.40
17	17752	2846.7	80.15	1.25 0.47 1.64 129.86 69.18 -28.27
18	17588	2256.1	309.52	2.10 -0.79 1.36 134.76 79.22 8.07
19	17588	2520.1	81.22	1.09 0.21 -0.11 126.86 88.54 -20.92
20	17348	2441.7	3.36	0.70 1.05 1.27 114.47 97.53 3.17

Table S5. Docking score of top 20 complexes of designed vaccine construct to TLR4 complexes generated by patchDock server

Solution No	Score	Area	ACE	Transformation
1	22972	4577.9	385.16	-1.38 0.44 1.65 17.60 20.70 -25.44
2	21532	3064.3	215.95	-0.73 -0.32 -0.33 -38.41 -14.52 40.15
3	21376	4205.6	-26.00	2.44 1.15 -2.23 -26.96 -28.12 19.72
4	21348	4023.7	-183.75	2.71 1.24 -2.66 -29.08 -26.38 24.55
5	21084	3362.3	356.74	-0.10 -1.09 0.35 -1.56 38.52 -37.94
6	19992	3107.8	433.65	-1.88 0.16 3.06 17.09 -9.48 -1.29

7	19166	3697.1	29.85	3.06 -0.75 -1.90 -43.23 22.68 -84.90
8	18956	4994.1	121.34	-1.98 -0.73 0.18 -1.03 -3.71 1.30
9	18850	2397.2	432.49	-0.71 -0.26 1.53 -58.22 14.00 -64.53
10	18684	2465.1	83.37	0.19 -1.24 2.22 -10.41 -20.61 43.09
11	18326	3234.7	299.37	-2.93 -0.72 0.87 -33.55 39.11 -18.27
12	18274	2713.2	177.82	0.56 0.44 -2.89 -44.58 8.31 -90.58
13	18158	3342.3	119.17	0.81 -0.69 -1.19 -24.52 45.28 -53.00
14	18106	2987	457.09	-0.85 1.11 2.84 28.31 -6.30 -24.38
15	17954	2829.7	-434.35	2.59 -0.53 -0.90 2.18 42.68 -18.59
16	17952	3254.7	-28.62	-1.37 -0.47 -2.16 -8.31 -36.40 20.15
17	17726	2769.6	402.10	-1.41 -0.67 0.21 -10.45 -18.79 -72.53
18	17702	2283.6	168.14	-2.40 0.61 -1.82 7.23 20.55 -90.44
19	17674	3475.9	494.22	0.64 -0.33 3.13 -28.23 17.66 11.30
20	17422	3831	233.01	-0.55 0.73 1.63 -7.23 -2.77 -81.27

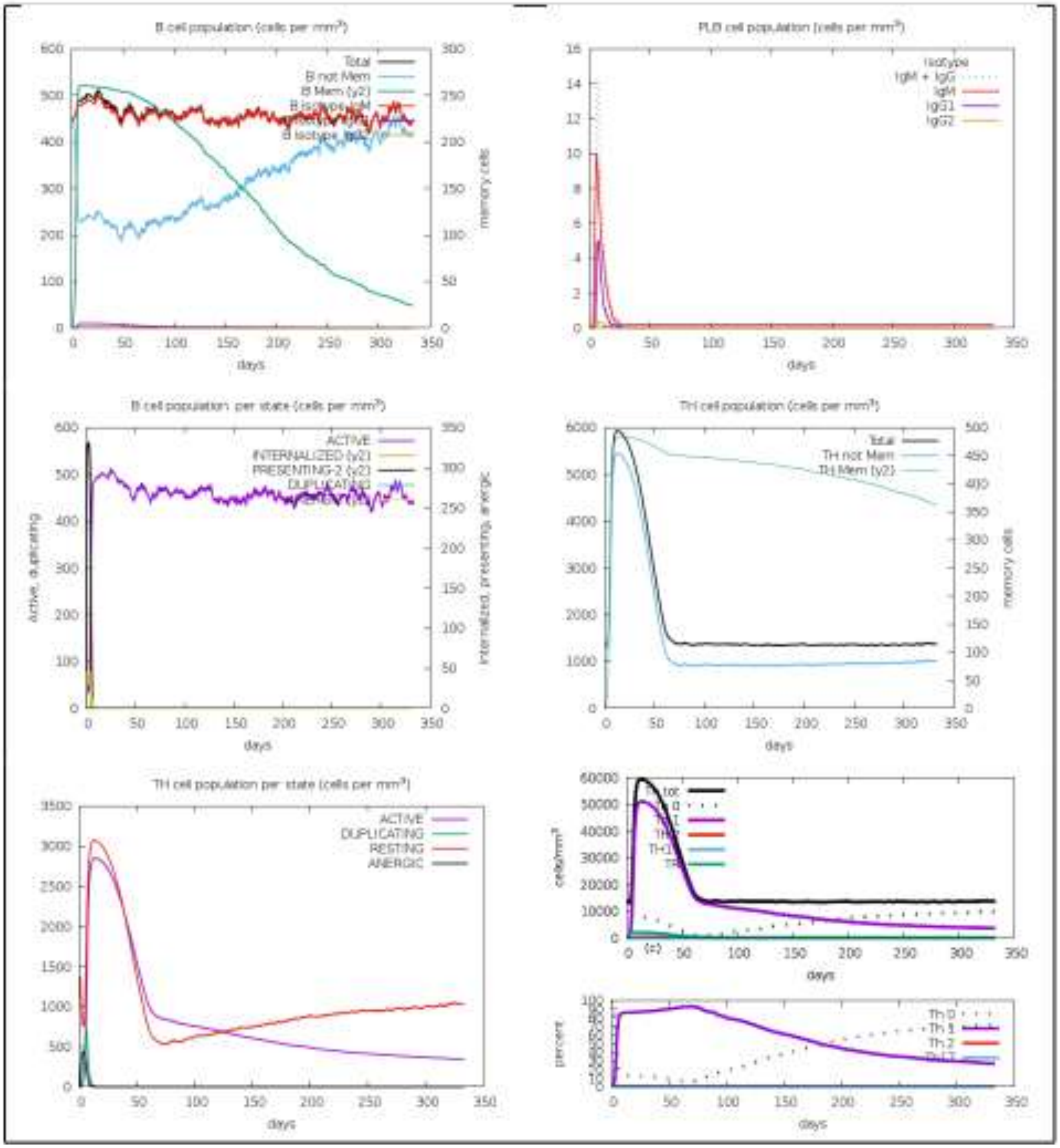

Figure S1. B cell and T cell population produced in response to the vaccine antigen.

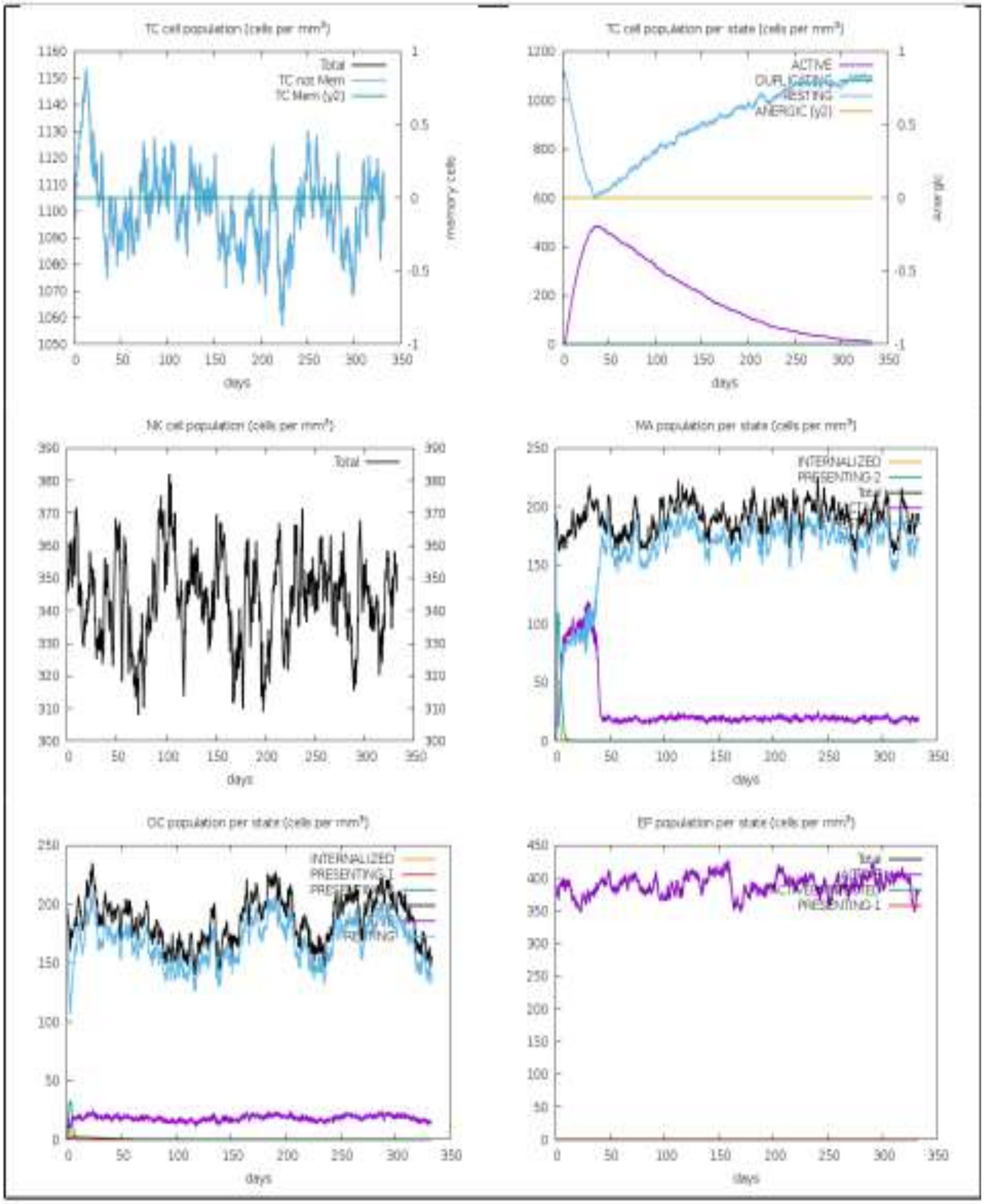

Figure S2. Immune cell response generated in response to chimeric vaccine construct. Tc (cytotoxic killer T-cell) Macrophages (Mφ) Nature killer cell, Dendritic and epithelial cell.
